# Extensive Intra-abdominal Desmoplastic Small Round Cell Tumor With Hepatic Metastases: A Report of a Rare Case

**DOI:** 10.7759/cureus.109298

**Published:** 2026-05-20

**Authors:** Zineb Guessous, Zakaria El Kodmiri, Mehdi Agal, Mounia Bendari, Maryame Ahnach

**Affiliations:** 1 Hematology Department, Cheikh Khalifa International University Hospital, Mohammed VI University of Health and Sciences (UM6SS), Casablanca, MAR; 2 Immunopathology-Immunotherapy-Immunomonitoring Laboratory, Faculty of Medicine, Mohammed VI University of Health and Sciences (UM6SS), Casablanca, MAR; 3 Department of Hematology, International University Hospital Mohammed VI, Rabat, MAR; 4 Faculty of Medicine, Mohammed VI University of Health and Sciences (UM6SS), Rabat, MAR

**Keywords:** abdominal mass, desmoplastic small round cell tumor (dsrct), ewsr1-wt1 fusion, malignancy, oncology, young adult

## Abstract

Desmoplastic small round cell tumor (DSRCT) is a rare and highly aggressive malignancy predominantly affecting young male patients, typically originating from the abdomen or pelvis, with a poor prognosis. Since the first description as a distinct clinical entity in 1989, approximately 850 cases have been reported in the literature. All cases share the chromosomal translocation t(11;22)(p13;q12), leading to the formation of the EWSR1-WT1 fusion gene. DSRCT characteristically arises in the abdomen or pelvis and carries a poor prognosis despite multimodal therapy. We report a rare and extensive case of abdominopelvic DSRCT with liver and peritoneal metastases in a 17-year-old Moroccan male, who presented with a progressive abdominal mass, pain, and hepatomegaly. The diagnosis was initially challenging due to the non-specific clinical presentation. The final diagnosis was established through liver biopsy and immunohistochemical analysis. The patient is currently undergoing treatment with a multidrug chemotherapy regimen.

## Introduction

Desmoplastic small round cell tumors (DSRCT) are rare sarcomas of mesenchymal origin. Sarcomas represent a small proportion of adult malignancies but occur more frequently in pediatric and adolescent populations. DSRCT is an exceptionally rare entity, with approximately 850 cases reported since its first description. It primarily affects adolescents and young adult males and most commonly arises in the abdominal cavity [1].

First described in 1989 by Gerald WL and Rosai J as a distinct clinicopathological entity, DSRCT is typically a peritoneal malignancy characterized by extensive intra-abdominal dissemination. It is defined by the t(11;22)(p13;q12) chromosomal translocation, which generates the EWSR1-WT1 fusion gene. This fusion protein functions as an abnormal transcription factor that promotes tumor development through dysregulated expression of genes involved in cellular proliferation and differentiation. DSRCT should be considered in the differential diagnosis of small round blue cell tumors, including Ewing sarcoma, rhabdomyosarcoma, lymphoma, and CIC-rearranged sarcoma. Accurate diagnosis requires integration of morphological, immunohistochemical, and, when available, molecular data [2].

Diagnosis is based on histomorphology showing polyphenotypic differentiation and on immunohistochemistry demonstrating variable epithelial, mesenchymal, and neural markers. Detection of the EWSR1-WT1 fusion gene confirms the diagnosis when available. No standardized treatment protocol exists, and management is based on multimodal approaches, including chemotherapy, surgery, and radiotherapy in specialized centers. Despite treatment, reported outcomes remain poor, with limited long-term survival [3].

The objective of this study is to highlight the diagnostic complexity and therapeutic challenges of DSRCT through a case of advanced intra-abdominal disease.

## Case presentation

A 17-year-old patient initially presented with an isolated painful abdominal mass, with no prior medical or surgical conditions. The patient reported a one-month history of right hypochondrial heaviness associated with vomiting, progressing in the context of general health deterioration.

A thorough clinical examination was supplemented by a contrast-enhanced multidetector thoraco-abdominopelvic CT scan, which revealed supradiaphragmatic and infradiaphragmatic lymph node involvement, peritoneal carcinomatosis, and secondary nodular hepatomegaly. Imaging identified a peritoneal mass with lobulated contours, originating from the mesenteric root, measuring 82 × 60 mm, and exhibiting extensive necrosis with scattered calcifications. The liver was enlarged and displayed multiple nodules and confluent masses, with peripheral enhancement following contrast administration. Lymph node assessment revealed mediastinal subcarinal adenopathies, as well as multiple necrotic lomboaortic, celiomesenteric, and retro-pancreatic lymph nodes, the largest measuring 30 × 25 mm (Figure 1).

**Figure 1:**
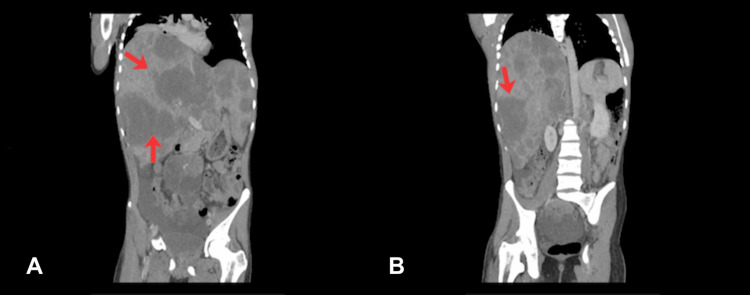
Thoraco-abdominal and pelvic CT scan demonstrating a large lobulated peritoneal mass arising from the mesenteric root (A), associated with secondary nodular hepatomegaly consistent with hepatic metastases (B), peritoneal carcinomatosis, and multiple lymphadenopathies involving the lomboaortic, celiomesenteric, retro-pancreatic, and mediastinal regions.

The biological workup revealed mild anemia with a hemoglobin level of 11.8 g/dL, while the white blood cell count remained within the normal range (5.04 ×10³/mm³). The inflammatory assessment showed an elevated C-reactive protein (CRP) level of 189.10 mg/L. Additionally, hyperuricemia was noted at 99 mg/L, along with an elevated lactate dehydrogenase (LDH) level of 1,758 IU/L. Liver function tests demonstrated cytolysis with elevated transaminases (AST and ALT). Mild electrolyte disturbances were also observed, including hyponatremia and decreased bicarbonate levels. The tumor biomarker alpha-fetoprotein (AFP) was negative, as were the serologies for hepatitis B, hepatitis C, and HIV (Table 1).

**Table 1:** Summary of key laboratory results RBC: Red blood cells; Hb: Hemoglobin; MCV: Mean corpuscular volume; WBC: White blood cells; PLT: Platelets; AST: Aspartate aminotransferase; ALT: Alanine aminotransferase; LDH: Lactate dehydrogenase; TP: Total proteins; Ca: Calcium; PT: Prothrombin time; aPTT: Activated partial thromboplastin time.

Parameters	Values	Reference range
RBC	4.58 ×10¹²/L	4.2–5.9 ×10¹²/L
Hb	11.8 g/dL	12.0–16.0 g/dL
MCV	78.8 fL	80–100 fL
WBC	5.04 ×10⁹/L	4.0–10.0 ×10⁹/L
PLT	454 ×10⁹/L	150–400 ×10⁹/L
Urea	0.29 g/L	0.15–0.45 g/L
Creatinine	5.52 mg/L	6–12 mg/L
AST	228 IU/L	10–40 IU/L
ALT	157 IU/L	7–56 IU/L
Uric acid	99 mg/L	25–70 mg/L
LDH	1758 IU/L	85–230 IU/L
Calcium	91 mg/L	88–105 mg/L
Total bilirubin	11 mg/L	2–12 mg/L
PT	100%	70–100%
aPTT	20.4 s	25–35 s
D-dimer	11.65 mg/L	< 0.5 mg/L

A liver biopsy was subsequently performed under sedation. Histopathological examination revealed a poorly differentiated tumor proliferation composed of nests, clusters, and cords of basaloid round cells, with a focal organoid architecture. The hyperchromatic round cells exhibited moderate atypia with occasional mitotic figures. Tumor necrosis was estimated at 30%. The stroma was abundant, desmoplastic or loose, with non-atypical spindle cells and fine vascularization.

Immunohistochemical analysis demonstrated positive heterogeneous staining with anti-CKAE1-AE, membranous positivity with anti-CD99, and intense diffuse dot-like positivity with anti-desmin. Anti-myogenin staining was negative, while the Ki-67 proliferation index was estimated at 45%. These findings were consistent with a diagnosis of DSRCT. The differential diagnosis included Ewing sarcoma, rhabdomyosarcoma, lymphoma, and CIC-rearranged sarcoma, which were excluded based on the immunohistochemical profile and morphological features. Molecular confirmation of the EWSR1-WT1 fusion transcript could not be performed due to the unavailability of molecular testing at our institution at the time of diagnosis (Figure 2).

**Figure 2:**
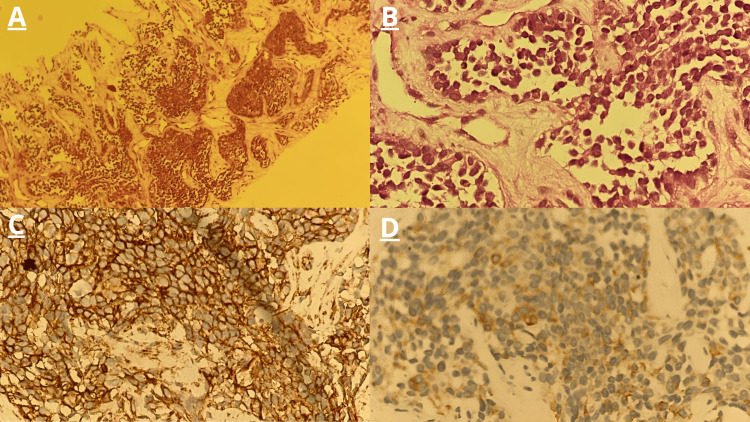
Histological and immunohistochemical examination of liver biopsy showing proliferation of small, round, basophilic cells, consistent with desmoplastic small round cell tumor. (A) Low-magnification view showing hepatic parenchyma infiltrated by nests and sheets of small round tumor cells (hematoxylin and eosin, ×10). (B) High-magnification view showing densely packed small round cells with hyperchromatic nuclei and scant cytoplasm (hematoxylin and eosin, ×40). (C) Immunohistochemistry showing positivity for cytokeratin (AE1/AE3) with cytoplasmic/membranous staining in tumor cells (×400). (D) Immunohistochemistry showing characteristic dot-like desmin positivity in tumor cells (×400).

The initial management consisted of the patient's hospitalization and stabilization. A first cycle of chemotherapy was administered according to the VIDE protocol, which included vincristine (1.5 mg/m², with a maximum dose of 2 mg), ifosfamide (3,000 mg/m²), doxorubicin (20 mg/m²), and etoposide (150 mg/m²) over a four-day period. This first treatment cycle resulted in significant clinical improvement, demonstrating a strong chemoresponsiveness, with a 16 cm reduction in abdominal circumference and a notable decrease in ascites. A subsequent cycle is scheduled at a 21-day interval.

## Discussion

DSRCT is a rare sarcoma characterized by a distinctive clinicopathological and molecular profile. Since its first description in 1989, approximately 850 cases have been reported in the literature. It predominantly affects adolescents and young adults, with a marked male predominance, and most commonly arises within the abdominal cavity [3,4].

Clinically, DSRCT typically presents as a large intra-abdominal mass associated with diffuse peritoneal dissemination, as observed in the present case. The absence of a specific organ of origin and extensive peritoneal involvement are important diagnostic features. Less frequently, extra-abdominal sites such as the pleura, mediastinum, bone, and central nervous system have been reported [5]. Symptoms are nonspecific, including abdominal pain, weight loss, vomiting, and occasionally ascites, often leading to delayed diagnosis [6].

Imaging plays a key role in initial evaluation. A contrast-enhanced CT scan is the first-line modality and typically shows a heterogeneous abdominal mass with peritoneal carcinomatosis, lymphadenopathy, and possible hepatic metastases. Thoracic imaging is recommended due to frequent metastatic spread. FDG-PET has demonstrated high lesion detection rates in small retrospective series and may improve staging accuracy; however, its clinical impact remains limited due to small sample sizes and lack of standardized outcome data [7,8].

Histopathology is essential for diagnosis. DSRCT is characterized by nests, cords, and clusters of small, round, blue cells embedded in a dense desmoplastic stroma. Tumor cells show hyperchromatic nuclei, scant cytoplasm, frequent mitoses, and areas of necrosis. Immunohistochemistry reveals a polyphenotypic profile with co-expression of epithelial markers (cytokeratin, EMA), mesenchymal markers (desmin with characteristic dot-like staining), and variable neural markers, while myogenin is consistently negative. CD99 expression may also be observed but is non-specific. This multilineage immunoprofile is a key diagnostic feature distinguishing DSRCT from other small round cell tumors [9,10].

The differential diagnosis includes Ewing sarcoma, rhabdomyosarcoma, lymphoma, CIC-rearranged sarcoma, and neuroblastoma. Although these entities may share similar morphological features, they were excluded in the present case based on the immunohistochemical profile, particularly the co-expression of cytokeratin and desmin with the absence of myogenin expression. Integration of morphology, immunophenotype, and clinical context is therefore essential for accurate diagnosis [11].

At the molecular level, DSRCT is defined by the t(11;22)(p13;q12) translocation resulting in the EWSR1-WT1 fusion gene, which acts as an aberrant transcription factor involved in tumorigenesis [12]. Although this alteration is considered a molecular hallmark of the disease, molecular confirmation was not performed in the present case due to the limited availability of diagnostic facilities, which represents a diagnostic limitation.

Despite multimodal treatment combining chemotherapy, surgery, and radiotherapy, prognosis remains poor, with five-year survival rates below 15% [7]. This poor outcome is mainly related to the advanced stage at diagnosis and extensive peritoneal dissemination. Management requires a multidisciplinary approach in specialized sarcoma centers to evaluate resectability and optimize therapeutic planning. Anthracycline-based chemotherapy regimens are commonly used, typically including doxorubicin.

These limitations in current outcomes have prompted ongoing research into novel therapeutic approaches, including targeted therapies and immunotherapy; however, current evidence remains preliminary and largely based on early-phase studies.

## Conclusions

This case highlights the diagnostic challenges associated with DSRCT and emphasizes the importance of maintaining a high index of suspicion for this rare malignancy in young patients. Diagnosis relies on characteristic morphological and immunohistochemical features, with molecular confirmation of the EWSR1-WT1 fusion when available; however, such confirmation was not performed in this case due to technical limitations.

Early recognition and prompt initiation of multidisciplinary management in specialized sarcoma centers are essential to optimize patient care. Although no standardized therapeutic protocol exists, treatment generally relies on a multimodal approach combining intensive chemotherapy, surgery, and, in selected cases, radiotherapy. The prognosis remains limited, underscoring the need for continued research into targeted therapies and improved treatment options.
